# Tsallis Entropy-Based Complexity-IPE Casualty Plane: A Novel Method for Complex Time Series Analysis

**DOI:** 10.3390/e26060521

**Published:** 2024-06-17

**Authors:** Zhe Chen, Changling Wu, Junyi Wang, Hongbing Qiu

**Affiliations:** 1 School of Information and Communication, Guilin University of Electronic Technology, Guilin 541004, China; chenzhe2015@guet.edu.cn (Z.C.); wcl1997@guet.edu.cn (C.W.); wangjy@guet.edu.cn (J.W.); 2 Cognitive Radio and Information Processing Key Laboratory Authorized by China’s Ministry of Education Foundation, Guilin University of Electronic Technology, Guilin 541004, China

**Keywords:** Tsallis entropy, improved permutation entropy, complexity-entropy casualty plane, feature extraction, time series analysis

## Abstract

Due to its capacity to unveil the dynamic characteristics of time series data, entropy has attracted growing interest. However, traditional entropy feature extraction methods, such as permutation entropy, fall short in concurrently considering both the absolute amplitude information of signals and the temporal correlation between sample points. Consequently, this limitation leads to inadequate differentiation among different time series and susceptibility to noise interference. In order to augment the discriminative power and noise robustness of entropy features in time series analysis, this paper introduces a novel method called Tsallis entropy-based complexity-improved permutation entropy casualty plane (TC-IPE-CP). TC-IPE-CP adopts a novel symbolization approach that preserves both absolute amplitude information and inter-point correlations within sequences, thereby enhancing feature separability and noise resilience. Additionally, by incorporating Tsallis entropy and weighting the probability distribution with parameter q, it integrates with statistical complexity to establish a feature plane of complexity and entropy, further enriching signal features. Through the integration of multiscale algorithms, a multiscale Tsallis-improved permutation entropy algorithm is also developed. The simulation results indicate that TC-IPE-CP requires a small amount of data, exhibits strong noise resistance, and possesses high separability for signals. When applied to the analysis of heart rate signals, fault diagnosis, and underwater acoustic signal recognition, experimental findings demonstrate that TC-IPE-CP can accurately differentiate between electrocardiographic signals of elderly and young subjects, achieve precise bearing fault diagnosis, and identify four types of underwater targets. Particularly in underwater acoustic signal recognition experiments, TC-IPE-CP achieves a recognition rate of 96.67%, surpassing the well-known multi-scale dispersion entropy and multi-scale permutation entropy by 7.34% and 19.17%, respectively. This suggests that TC-IPE-CP is highly suitable for the analysis of complex time series.

## 1. Introduction

The concept of entropy was first introduced by Clausius in 1854 as a measure of disorder in thermodynamic systems. In 1948, Shannon extended this concept to the field of communications by proposing information entropy, thereby enabling the quantification of information [1]. In recent years, entropy has garnered increasing attention and has been widely applied in various fields [2,3,4], including system identification, time series analysis, and signal recognition.

This paper focuses on the application of entropy in time series analysis. Currently, entropy algorithms used to measure the randomness of time series are primarily based on the definitions of conditional entropy and Shannon entropy. A notable example of the former is approximate entropy (ApEn), proposed by Pincus in 1991 [5]. ApEn evaluates the similarity between sequences in phase space, effectively measuring the complexity of time series. However, ApEn has significant drawbacks, including a strong dependence on the length of the time series and relatively high error rates. In 2002, Richman and colleagues improved upon ApEn and proposed sample entropy (SampEn), which addresses the self-matching issue in ApEn and enhances algorithm stability [6]. Chen and co-researchers argued that SampEn’s use of the Heaviside function, a hard threshold method for measuring sequence similarity, leads to inaccurate entropy estimation. To mitigate this, they replaced the Heaviside function with a fuzzy membership function, improving the algorithm’s performance. Despite these advancements, all three types of algorithms are affected by the tolerance factor r and have high computational complexity, making them unsuitable for real-time processing.

A notable representative of entropy algorithms based on Shannon entropy is permutation entropy (PE) [7], proposed by Bandt in 2002. PE utilizes “symbol patterns” to characterize time series, thereby reducing the computational complexity of the algorithm. This approach has seen widely applied across various fields. However, PE neglects amplitude information, leading to a diminished ability to distinguish between different time series. Although subsequent improvements such as weighted permutation entropy [8] and modified permutation entropy [9] have been proposed, these methods fail to simultaneously consider both the absolute amplitude information of sample points and the temporal correlation between them. In 2016, Mostafa and colleagues introduced the dispersion entropy (DispEn) algorithm [10], which not only offers fast computation but is also less affected by abrupt signal changes. In 2018, Azami and colleagues further refined DispEn by incorporating signal fluctuations, resulting in fluctuation dispersion entropy (FDispEn) [11], which exhibits enhanced noise resistance compared to DispEn. In addition, methods such as bubble entropy, distribution entropy, slope entropy, and attention entropy can measure the randomness of time series from different perspectives; however, none of these methods have been as widely applied in practice as PE and DispEn.

All above mentioned entropy algorithms operate at a single scale, providing a solitary entropy estimate for a given time series. However, in practical engineering scenarios, time series generated by complex systems exhibit inherent complexity that cannot be adequately captured by single-scale entropy features. Recognizing this challenge, Costa proposed a coarse-graining technique to decompose the original series into sequences at various scales for multi-scale entropy estimation [12]. This approach, known as multi-scale entropy estimation, enables a more comprehensive understanding of the system’s complexity. The entropy values obtained at different scales are then integrated into a feature vector. Through the integration of coarse-graining techniques with entropy algorithms, scholars have introduced a range of multi-scale entropy algorithms, such as multi-scale permutation entropy (MPE) [13], multi-scale sample entropy [12], and multi-scale dispersion entropy (MDispEn) [14]. The adoption of this coarse-graining strategy significantly enhances the discriminative power of entropy features across diverse signal types.

Entropy measures serve as effective tools for quantifying the randomness and disorder present in time series data. However, they often fall short in capturing the underlying structural correlations that are intricately woven within these sequences, thus failing to provide a comprehensive characterization of time series complexity. In 1995, Lopez, Mancini, and Calbert proposed a novel measure of complexity known as statistical complexity (SCM) [15]. SCM is derived by multiplying the imbalance of probability density distribution by the information entropy. Initially, SCM utilized histogram and bisection methodologies to depict the probability density distribution of time series. Subsequently, researchers amalgamated algorithms such as Shannon entropy and permutation entropy to devise the complexity-entropy causality space (CECS). Zhang et al. conducted financial data analysis utilizing complexity-entropy methodologies [16], while Li et al. employed similar techniques to investigate the dynamic characteristics of gas–liquid two-phase flow [17]. Silva et al. applied CECS to examine monthly rainfall time series. These studies collectively underscore the efficacy of CECS. However, it is noteworthy that the majority of these entropy computations rely on Shannon entropy or permutation entropy, thereby inheriting the limitations associated with amplitude information loss and inadequate handling of equal elements within embedding vectors.

This study introduces a Tsallis entropy-based complexity-improved permutation entropy casualty plane (TC-IPE-CP) to address challenges encountered in traditional entropy algorithms during the extraction of features from complex time series, including substantial data point requirements, limited separability, and weak noise resilience. Departing from conventional complexity-permutation entropy planes, this method adopts a novel symbolization approach that preserves both absolute amplitude information and inter-point correlations within sequences, thereby enhancing feature separability and noise resilience. Additionally, by incorporating Tsallis entropy and weighting the probability distribution P with parameter q [18,19], it integrates with statistical complexity to establish a feature plane of complexity and entropy, further enriching signal features. Through the integration of multiscale algorithms, a multiscale Tsallis-improved permutation entropy algorithm is also developed.

The paper’s structure is outlined as follows: Section 2 delineates the fundamental principles of TC-IPE-CP, Section 3 and Section 4 offer analyses of simulated signals and complex time series utilizing various multiscale entropy algorithms, and Section 5 furnishes a comprehensive summary of the paper’s entirety.

## 2. Methods

### 2.1. Tsallis Improved Permutation Entropy (TIPE)

The procedural delineation of the TIPE algorithm unfolds as follows:

Step 1. For a time series X={$x_{1},x_{2},\ldots ,x_{N}$}, normalization is performed by employing Equation (1) for the cumulative distribution function, resulting in the normalized sequence, denoted as $y_{i}$. In the equation, μ and $\delta^{2}$, respectively, represent the mean and variance.
  (1) $$y_{i}=\frac{1}{\delta \sqrt{2\pi }}\int_{-\infty }^{x_{i}}e^{\frac{{-\left(t-\mu \right)}^{2}}{2\delta^{2}}}dt$$
Step 2. By setting the embedding dimension *m* and time delay τ, the time series is reconstructed as follows:
  (2) $$Y_{j}=\left[y_{j},y_{j+\tau },\ldots ,y_{j+\left(m-1\right)\tau }\right],$$
  where $Y_{j}$ represents the *j*-th row of Y, and $1\ll j\ll N-\left(m-1\right)\tau$.
Step 3. By applying the uniform quantization operator (UQO) as defined in Equation (3), the first column Y(:,1) is transformed into S(:,1), representing the first column of the symbolized phase space.
  (3) $$UQO\left(u\right)=\left\{\begin{array}{c} 0\\ 1\\ \ldots \\ L-1 \end{array}\right.\begin{array}{c} y_{min}\leq \mu <\Delta +y_{min}\\ y_{min}+\Delta \leq \mu <2\Delta +y_{min}\\ \ldots \\ y_{max}-\Delta <\mu \leq y_{max} \end{array}$$
  In Equation (3), $y_{max}$ and $y_{min}$ are, respectively, the maximum and minimum values of y, L is the predetermined discretization parameter, and Δ represents the discrete interval, which satisfies Δ = ($y_{max}$ − $y_{min}$)/L.
Step 4. Define Y(:,k) as the k-th column of Y, where 2≪k≪m. Symbolize Y(:,k) using Equation (4) to obtain S(:,k).
  (4) $$S\left(j,k\right)=S\left(j,1\right)+\left\lfloor \left(Y\left(j,k\right)-Y\left(j,1\right)\right)/\Delta \right\rfloor ,$$
  where $1\leq j\leq N-\left(m-1\right)\tau$, and ⌊⌋ represents the floor function, which means rounding down to the nearest integer. After symbolizing all components of Y, the resulting entity is represented as the symbolic phase space S. Furthermore, each row of S is referred to as a symbolic pattern (SP), which incorporates both permutation relations and amplitude information. According to Equation (4), after symbolization, each pattern contains m elements, with each element having L possible states. Hence, the total number of symbolic patterns is $L^{m}$.
Step 5. In contrast to other approaches, here we employ Tsallis entropy to compute entropy values, which is a generalized form of Shannon entropy.
  (5) $$S_{q}\left(P\right)=\sum_{j=1}^{L^{m}}P_{j}\ln_{q}\frac{1}{P_{j}},$$
  where $\ln_{q}x=\frac{x^{1-q}-1}{1-q}$ and $q\neq 1,\ln_{1}x=\ln x\;\mathrm{for}\;\mathrm{any}\;x>0$, q represents the weighting coefficient. When the probability distribution follows a uniform distribution, the maximum value of Tsallis q entropy is $S_{q}\left(U\right)={ln}_{q}L^{m}({U=\{1/L^{m}\}}_{j=1,\ldots \ldots ,L^{m}}$), and L represents a pre-defined discretization factor. The normalized TIPE can be obtained as follows:
  (6) $$H_{q}[P]=\frac{S_{q}\left(P\right)}{S_{q}\left(U\right)}=\frac{\sum_{j=1}^{L^{m}}P_{j}\ln_{q}\frac{1}{P_{j}}}{{ln}_{q}L^{m}}$$

### 2.2. Multiscale Tsallis-Improved Permutation Entropy

In the analysis of intricate time series, it is customary to employ multiple temporal scales to assess the sequence’s complexity, a feature that surpasses the explanatory capacity of traditional entropy measures reliant solely on single-scale analysis. Therefore, we propose the concept of multiscale Tsallis improved permutation entropy (MTIPE). The time series $X=\{x_{1},x_{2},\ldots ,X_{n}\}$ undergoes a coarse-graining process [12] according to Equation (7) resulting in the output sequence $y_{j}^{S}$ at scale *s*. The sequence $y_{j}^{S}$ is input into the IPE algorithm to compute entropy values at scale *s*.

(7) $$y_{j}^{S}=\frac{1}{s}\sum_{i=\left(j-1\right)s+1}^{js}x_{i},1\leq j\leq N/s$$

### 2.3. TC-IPE-CP

Complexity entropy casualty plane (CECP) has been demonstrated to have certain advantages in revealing the causal relationships between internal structure and behavior within systems [20,21,22,23]. But the CECP based on the permutation entropy algorithm inherits its limitations. We propose a complexity-IPE causality plane based on the Tsallis entropy.

The complexity measure adopts Jensen–Shannon divergence to assess the imbalance between probability distributions and uniform distributions, which is the same as CECP. The calculation steps of TC-IPE-CP are as follows:

Step 1. Define the imbalance $\Delta_{q}[P]$ of the probability density distribution P according to Equation (8).
  (8) $$\Delta_{q}\left[P\right]=\frac{D_{q}\left(P,U\right)}{D_{q}^{*}\left(P,U\right)}$$
  where ${U=\{1/L^{m}\}}_{j=1,\ldots ..,L^{m}}$ represents the uniform distribution, and $D_{q}(P,U)$ represents the distance between the probability density distribution P and the uniform distribution U. $D_{q}^{*}\left(P,U\right)=\max D_{q}\left(P,U\right)$ is the maximum Jensen–Shannon divergence (JSD) distance. The calculation of $D_{q}(P,U)$ is shown in Equation (9).
  (9) $$D_{q}\left(P,U\right)=\frac{1}{2}{Κ}_{q}\left(P|\frac{P+U}{2}\right)+\frac{1}{2}{Κ}_{q}\left(U|\frac{P+U}{2}\right)\;\;=-\frac{1}{2}\sum_{\begin{array}{c} j=1\\ P_{j}\neq 1 \end{array}}^{L^{m}}P_{j}\ln_{q}\frac{P_{j}+\frac{1}{L^{m}}}{2P_{j}}--\frac{1}{2}\sum_{j=1}^{L^{m}}\frac{1}{L^{m}}\ln_{q}\frac{{(P}_{j}+\frac{1}{L^{m}})L^{m}}{2}\;$$
Step 2. Multiplying the normalized Tsallis entropy $H_{q}[P]$ by the imbalance $\Delta_{q}[P]$ yields the following complexity:
  (10) $$C_{q}[P]=\Delta_{q}\left[P\right]\cdot H_{q}\left(P\right)=\frac{D_{q}\left(P,U\right)\cdot H_{q}\left(P\right)}{D_{q}^{*}\left(P,U\right)}$$

Plotting $H_{q}\left(P\right)$ as the horizontal axis and $C_{q}\left(P\right)$ as the vertical axis, we can obtain the TC-IPE-CP feature. Combining the coarse-graining process defined by Equation (7), different scales of TC-IPE-CP can be obtained. The multiscale version of TC-IPE-CP is expressed as MTC-IPE-CP in the following.

## 3. Synthetic Data Analysis

In this section, TC-IPE-CP is employed to analyze several different simulated signals. As indicated by the analysis in Section 2, this algorithm requires appropriate parameter settings, including embedding dimension *m*, discretization parameter L, parameter factor *q*, and time delay τ. According to the conclusions of the paper [24], the range of *m* from 3 to 7 and a time delay *τ* = 1 are considered to yield better results. Unless explicitly stated otherwise, we set the discretization parameter L in the range of 2 to 8, m = 4, *τ* = 1, and q ranges from 0 to 100 with a step size of 0.001.

### 3.1. Noise Signals

Noise in time series analysis can significantly impact the predictability of data and the accuracy of models. White noise and pink noise are two common types of noise in time series analysis, characterized by their power spectral density distributions represented as $S_{W}\left(f\right)=C_{W}$ and $S_{P}\left(f\right)=C_{P}/f$, where $C_{W}$ and $C_{P}$ are constants.

**Figure 1:**
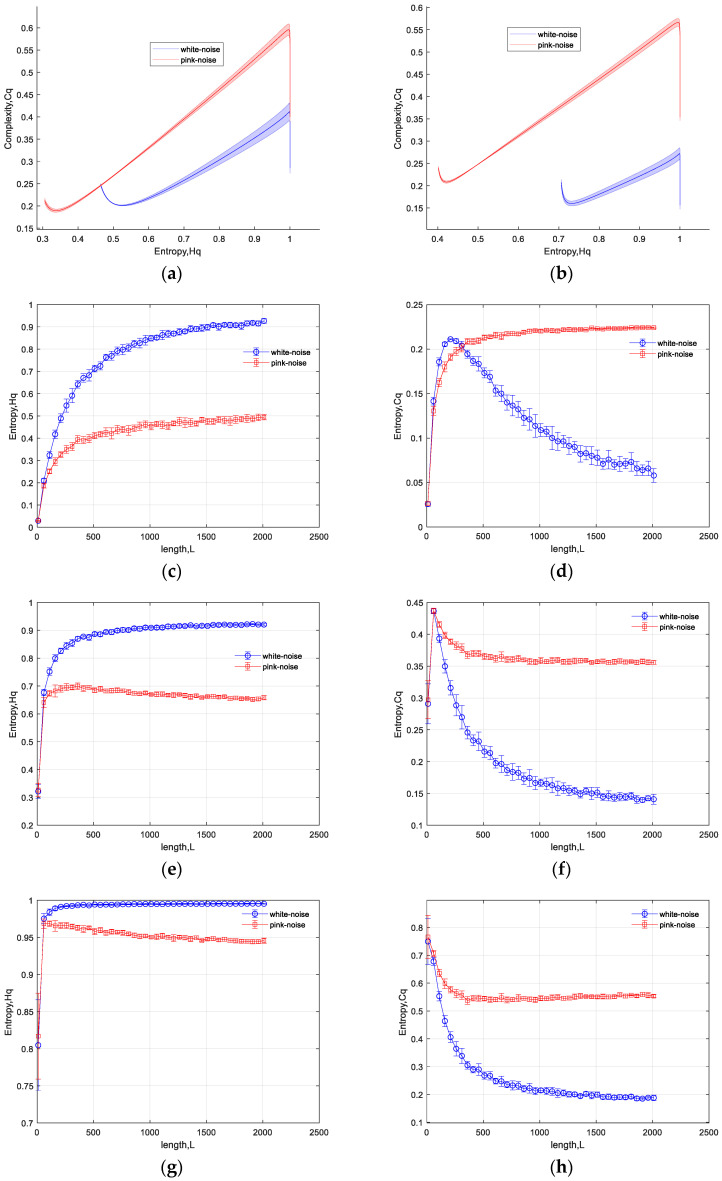
The TC-IPE-CP for different lengths of white noise and pink noise judgment results. (**a**) $H_{q}$-$C_{q}$ curves with L = 210; (**b**) $H_{q}$-$C_{q}$ curves with L = 510; (**c**) error bar plot of $H_{q}$ with q = 0.1; (**d**) error bar plot of $C_{q}$ with q = 1.1; (**e**) error bar plot of $H_{q}$ with q = 1.01; (**f**) error bar plot of $C_{q}$ with q = 1.01; (**g**) error bar plot of $H_{q}$ with q = 2.01; and (**h**) error bar plot of $C_{q}$ with q = 2.01.

In order to validate the reparability and reliability of the TC-IPE-CP under short time series conditions, we vary the data length L from 10 to 2010 with a step size of 50 sample points. For each data length, 20 sets of independent noise are generated. The results are depicted in the Figure 1 below. Figure 1a,b depicts the TC-IPE-CP characteristics of white noise and pink noise for data lengths of 210 and 510, respectively. The standard deviation of the results from multiple experiments is represented by shaded regions. In the Figure 1a, the blue curve represents the characteristic of white noise, where entropy $H_{q}$ decreases initially to 0.46 with increasing q, and then increases to 1; and complexity $C_{q}$ decreases initially to 0.2 with increasing q, and then increases to 0.43. The trend of $H_{q}$ $C_{q}$ varying with the parameter q forms a curve on the plane. The red curve corresponds to the characteristic of pink noise, similarly forming a curve on the plane. From the distribution of the characteristic curves on the plane, it is easy to distinguish between these two types of noise. Furthermore, the observed consistency across multiple experiments, as indicated by the small magnitude of the shaded areas, underscores the algorithm’s robust stability. For a more comprehensive display of the influence of data length on entropy $H_{q}$ and complexity $C_{q}$, we present curves depicting the variations of $H_{q}$ and $C_{q}$ with increasing length L, where *q* = 0.1, 1.01, and 2.01. The figures clearly demonstrate that classification of the two types of noise signals is feasible even when the length *L* is less than 100.

### 3.2. Autoregressive (AR) Time Series

AR model is a process in which regression variables are derived from the variable itself. It describes a linear regression model of a random variable at a future time by incorporating a linear combination of random variables from previous time steps. AR processes can be generated by Equation (11).

(11) $$AR_{p}\left(t\right)=\sum_{i=1}^{p}\alpha_{i}AR\left(t-i\right)+n(t)$$

where n(t) is white Gaussian noise (WGN) with zero mean and unit variance, p denotes the order of the AR sequence, when p = 0 implies that $AR_{0}$ is equivalent to WGN. The $\alpha_{i}$ represents autocorrelation coefficients. The parameter configurations for AR time series of varying orders are outlined in Table 1. Typically, a higher order of AR corresponds to increased sample correlation, enhanced predictability, and diminished randomness within the sequence.

**Figure 2:**
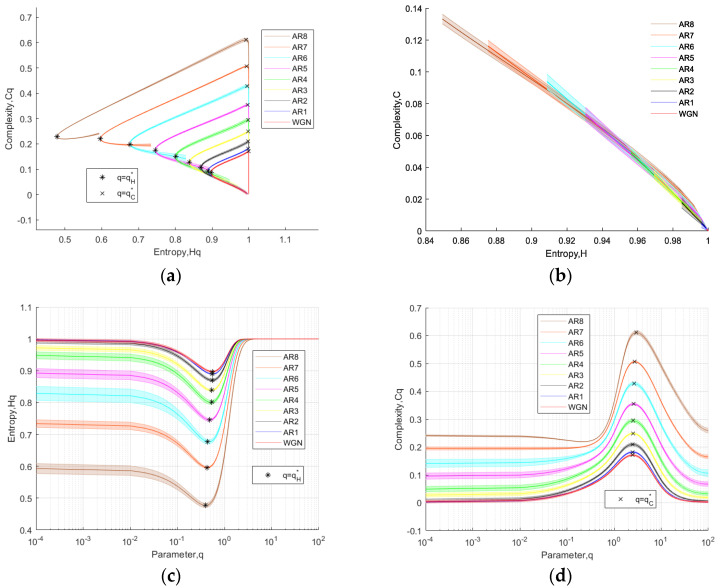
The analysis results of 20 sets of autoregressive time series and white noise on TC-IPE-CP and RC-PE casualty plane. (**a**) $H_{q}$-$C_{q}$ curves of TC-IPE-CP; (**b**) H-C curves of RC-PE-CP; (**c**) q-$H_{q}$ curves of TC-IPE-CP; and (**d**) q-$C_{q}$ curves of TC-IPE-CP.

Following the reference [25], we conducted feature analysis on AR processes of different orders using the TC-IPE-CP. We conducted independent analysis experiments on 20 sets of AR sequences ranging from order one to eight and WGN, with the length of the AR set to *N* = 10,000. The results are illustrated in Figure 2. Figure 2a depicts the results of 20 independent experiments conducted using TC-IPE-CP on AR sequences of eight different orders and WGN, and the shaded areas represent the standard deviation. In Figure 2a, the $H_{q}$-$C_{q}$ characteristic curves for AR sequences, ranging from AR one to eight, systematically expand outward. Additionally, the clear differentiation between WGN and the eighth AR signals underscores the algorithm’s ability to effectively discriminate signals with diverse dynamical characteristics. Figure 2b presents the results of experiments on 20 sets of AR sequences using Renyi complexity permutation entropy casualty plane (RC-PE-CP) (with alpha values ranging from 0.001 to 4.01 with a step size of 0.001), keeping other parameters consistent with TIPE. By comparing Figure 2a,b, which represent two types of complexity-entropy plane methods, it can be concluded that the TC-IPE-CP exhibits superiority in discriminating AR signals.

Following the analysis method outlined in reference [19], we identify the characteristic point $q_{H}^{*}$ corresponding to the minimum value of $H_{q}$ for each signal type, which is annotated as a black ∗ in the figure. Similarly, the characteristic point $q_{C}^{*}$, representing the maximum value of $C_{q}$ for each signal type, is identified and annotated as a black × in the figure. It is evident that there are significant differences in $q_{H}^{*}$ and $q_{C}^{*}$ across each signal type, suggesting this as a distinguishing feature. To validate this point, we plot the *q*-$H_{q}$ curve in Figure 2c and the *q*-$C_{q}$ curve in Figure 2d, while simultaneously annotating the positions of $q_{H}^{*}$ and $q_{C}^{*}$. It is evident that utilizing $q_{H}^{*}$ and $q_{C}^{*}$ provides effective discrimination for each type of AR signal and WGN.

### 3.3. Noisy Lorenz Signal

**Figure 3:**
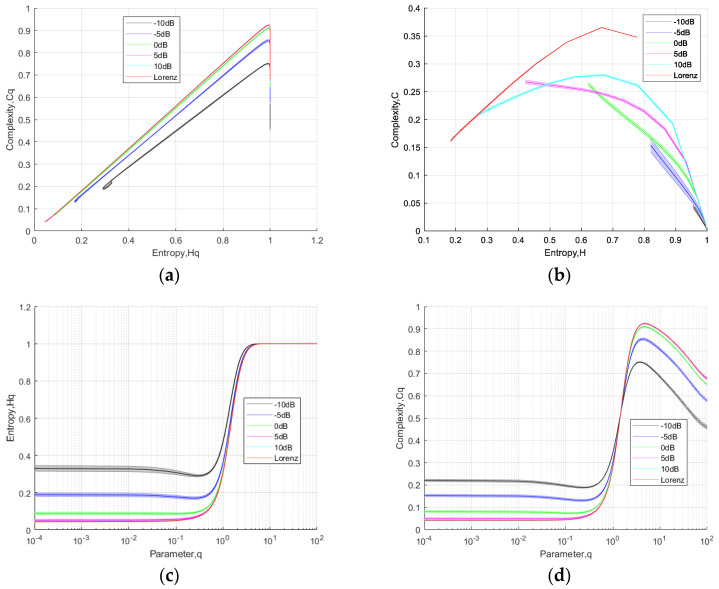
The analysis results of TC-IPE-CP and RC-PE-CP under different signal-to-noise ratio conditions for the Lorenz time series: (**a**) $H_{q}$-$C_{q}$ curves of TC-IPE-CP; (**b**) H-C curves of RC-PE-CP; (**c**) q-$H_{q}$ curves of TC-IPE-CP; and (**d**) q-$C_{q}$ curves of TC-IPE-CP.

In this section, we assess the algorithm’s performance under noisy conditions by generating signals at different SNR through the introduction of Gaussian white noise into the Lorenz time series. We solve the Lorenz system represented by Equation (12) using the fourth-order Runge–Kutta scheme with a time step of Δ*t* = 0.001, recording 50,000 data points. For each signal-to-noise ratio condition, we conducted 20 independent experiments using both TC-IPE-CP and RC-PE casualty plane, and calculated the entropy and complexity. From the results in Figure 3a, it can be observed that the curves of $H_{q}$ and $C_{q}$ under different (SNR) conditions for Lorenz signals in the TC-IPE-CP exhibit a high degree of similarity. Moreover, the curve for −10 dB closely resembles the curve for the normal signal, indicating that noise has the least impact on the performance of the TC-IPE-CP. Furthermore, the trends of the *q*-$H_{q}$ and *q*-$C_{q}$ curves for Lorenz chaotic signals under different SNR in Figure 3c,d are largely consistent, providing additional evidence of the superior noise resistance performance of the TC-IPE-CP. In comparison, the curves for RC-PE-CP of Lorenz signals under different SNR in Figure 3b exhibit relatively scattered patterns.

(12) $$\left\{\begin{array}{c} \dot{x}=10(y-x)\\ \dot{y}=x\left(28-z\right)-y\\ \dot{z}=xy-\frac{8}{3}x \end{array}\right.$$

## 4. Experimental Data Analysis

In this section, we validate the algorithm’s performance in handling real complex time series by conducting data analysis on experimental data of RR intervals, bearing fault signals, and acoustic signals using TC-IPE-CP.

### 4.1. RR Intervals

This section analyzes the RR intervals data from the Fantasia dataset [26], which includes data from young and elderly healthy participants. The age range of the young participants is 21 to 34 years, while the age range of the elderly participants is 68 to 85 years.

**Figure 4:**
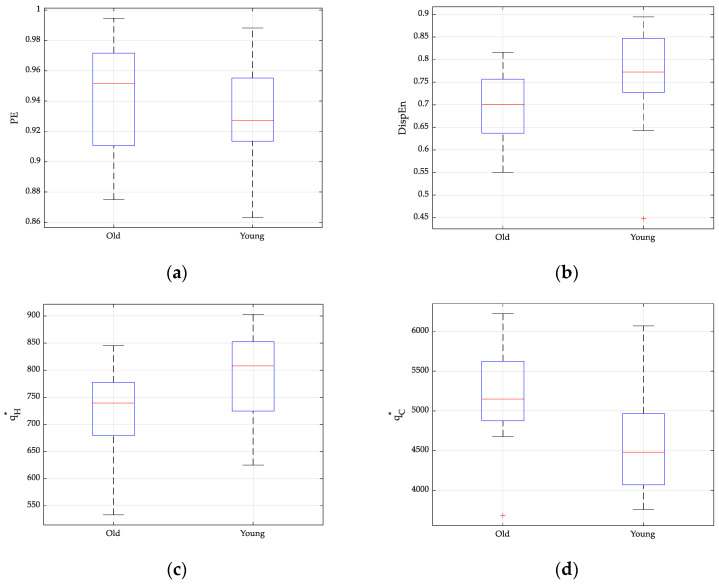
Boxplots of distinct entropy approaches computed from the RR intervals of healthy young and healthy elderly participants. (**a**) PE analysis result; (**b**) DE analysis result; (**c**) $q_{H}^{*}$ analysis result; and (**d**) $q_{C}^{*}$ analysis result. The symbol + in this figure represents outlier value.

To quantitatively assess the differences in entropy values between young and elderly individuals, we used the non-parametric Mann–Whitney U test. This method determines the significance of group differences through the *p*-value, with a lower *p*-value indicating more significant differences. The analysis results in Figure 4a,b are derived from the author’s article [27], where the *p*-value for PE is 0.2792 and the *p*-value for DispEn is 0.0038. In Figure 4c,d, the same dataset is processed using TC-IPE-CP. The analysis, using the $q_{H}^{*}$ and $q_{C}^{*}$ proposed in this paper, yields *p*-values of 0.0173 and 0.0022 for $q_{H}^{*}$ and $q_{C}^{*}$, respectively. In Figure 4, *p*-values smaller than 0.01 and 0.001247 are represented by ** and ***, respectively. The *p*-value for PE is 0.2792, which is greater than 0.01. The *p*-values for DispEn, $q_{H}^{*}$, and $q_{C}^{*}$ are 0.0038, 0.0173, and 0.0022, respectively, all falling between 0.01 and 0.001. They can all be represented by **, providing strong evidence for their ability to distinguish between two signal types.

### 4.2. Bearing Fault Signals

In this section, we selected four types of signals for analysis: normal signals with a fault diameter of 0.5334 mm and a motor speed of 1750, as well as signals representing rolling element faults, inner race faults, and outer race faults. Each dataset consisted of 120,000 data points. The dataset consists of four types of signals: normal signals, representing ball faults (BFs), inner race faults (IRFs), and outer race faults (ORFs) [28]. For ease of analysis, each dataset was divided into 10 segments, each containing 12,000 sample points. The bearing fault data analyzed was sourced from the Bearing Data Center at Case Western Reserve University.

**Figure 5:**
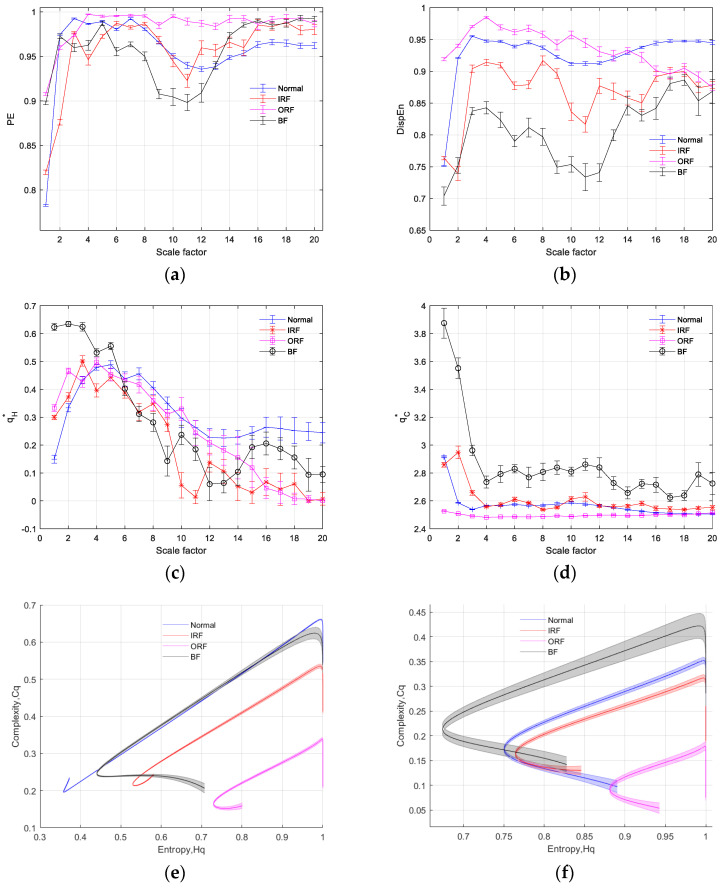
Multiscale entropy analysis results of four types of bearing fault signals. (**a**) PE analysis result; (**b**) DE analysis result; (**c**) $q_{H}^{*}$ analysis result; (**d**) $q_{C}^{*}$ analysis result; (**e**) $H_{q}$-$C_{q}$ curves of TC-IPE-CP with scale = 1; and (**f**) $H_{q}$-$C_{q}$ curves of TC-IPE-CP with scale = 5. (Note: In this paper, $q_{H}^{*}$ refers to the value of q when the entropy is at its maximum, and $q_{C}^{*}$ refers to the value of q when the complexity is at its maximum. These will not be annotated further in subsequent sections).

The results of multiscale entropy analysis for the four types of bearing fault signals are depicted in Figure 5. In Figure 5a, in the multiscale PE results, the entropy curves appear to be quite close to each other. In Figure 5b, the multiscale DispEn results show that the entropy information of the four types of faults is well separated from each other. In Figure 5c,d, it can be observed that the feature curves of $q_{H}^{*}$ and $q_{C}^{*}$ in the MTC-IPE-CP algorithm are well separated from each other. To demonstrate this point, Figure 5e and Figure 5f, respectively, depict the characteristic curve plots of $H_{q}$-$C_{q}$ for different fault information at scales one and five. From the distribution of curves on the plane, it is easy to distinguish between different types of faults.

### 4.3. Underwater Acoustic Signals

In this section, the MTC-IPE-CP is utilized to analyze four types of underwater acoustic signals, i.e., ambient noise, passenger boats, ocean liners, and motorboats [29]. The traditional multiscale PE and multiscale DispEn are also used for comparison purpose. Due to the large volume of data, each type of signal is segmented into multiple samples with a length of 3 s. The sampling frequency is set to 52,734 Hz, with each sample consisting of 158,202 data points. Table 2 provides detailed information about the datasets used in this experiment, which include four types of signals. Each class of data involves signals collected from different vessels.

**Figure 6:**
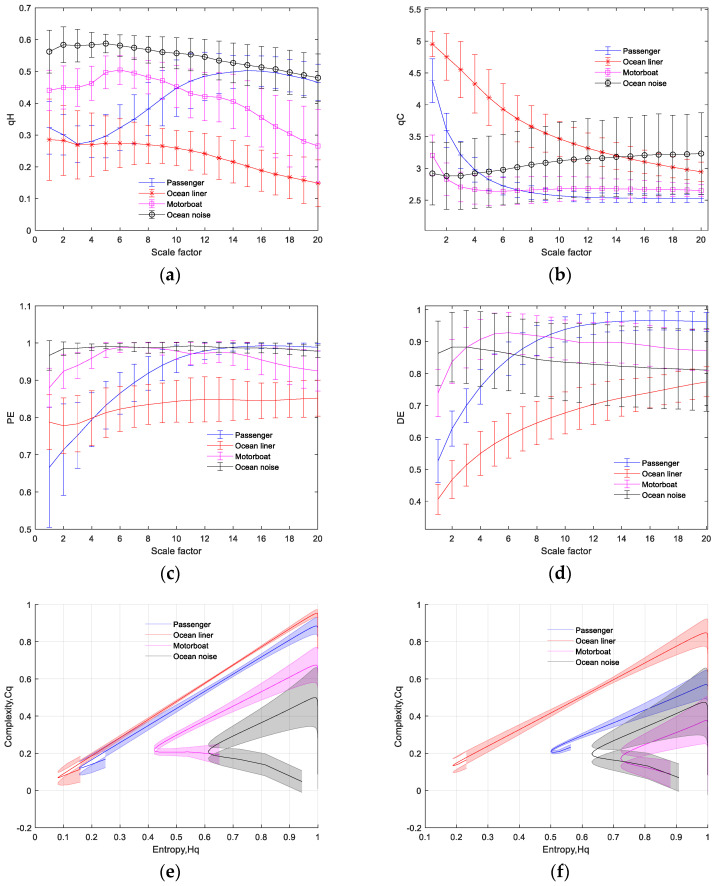
Multiscale entropy analysis results of four types of ship-radiated noise. (**a**) $q_{H}^{*}$ analysis result; (**b**) $q_{C}^{*}$ analysis result; (**c**) PE analysis result; (**d**) DE analysis result; (**e**) $H_{q}$-$C_{q}$ curves of TC-IPE-CP with scale = 1; and (**f**) $H_{q}$-$C_{q}$ curves of TC-IPE-CP with scale = 5.

The analysis results of multiscale PE, multiscale DispEn, and MTC-IPE-CP with a scale factor of 20 are depicted in Figure 6. As shown in Figure 6a, the three types of underwater acoustic signals, ocean liners, ocean noise, and motorboats, can be easily distinguished based on the magnitude of $q_{H}^{*}$ values at different scales. We also noticed with surprise that the trend of passenger $q_{H}^{*}$ decreases and then increases with the increase in scale, which is distinctly different from the other three types of signals. In Figure 6b, the $q_{C}^{*}$ values of ocean liners, passenger boats, and motorboats exhibit a decreasing trend followed by stabilization with the increase in scale. Additionally, there is a significant difference in the magnitude of $q_{C}^{*}$ values between scale one and five for these three types of ships. Unlike the others, ocean noise exhibits relatively minor changes in $q_{C}^{*}$ values as the scale increases. The results of multiscale PE are shown in Figure 6c, where it can be observed that as the scale increases, the curves of ocean liners, ocean noise, and motorboat PE exhibit significant overlap. In Figure 6d, which depicts the results of multiscale DispEn, there are distinct overlapping regions in entropy values between ocean noise and motorboats at different scales. In addition, Figure 6e,f presents the $H_{q}$-$C_{q}$ feature curves at scales one and five, respectively. From the distribution of curves on the plane, it is straightforward to distinguish between the four types of underwater acoustic signals. The above results demonstrate that compared to multiscale PE and multiscale DispEn, MTC-IPE-CP shows greater potential for classifying the four types of underwater acoustic signals.

To further substantiate this, we trained and recognized the features extracted by TC-IPE-CP using a probabilistic neural network (PNN), quantifying the algorithm’s discriminative efficacy. It is worth noting that we combined the two features of MTC-IPE-CP, $q_{H}^{*}$ and $q_{C}^{*}$, before classifying using PNN. During testing, we divided the training and testing sets for PNN. We selected 150 segments for each type of underwater acoustic signal as the testing set, and the remaining segments were used for training. The classification results of TC-IPE-CP for the four types of underwater acoustic signals are presented in Table 3. Additionally, we provide the classification results of MPE and multiscale DispEn algorithms in Table 4 and Table 5, respectively.

The results from the three tables indicate that MTC-IPE-CP, multiscale DispEn algorithm, and multiscale PE all achieved impressive recognition rates of 100% for the ocean liner category. However, in the passenger category, MTC-IPE-CP achieved a recognition rate of 96.67%, which is higher than the other two methods. Moreover, the algorithm’s classification performance for the four types of vessels exceeded 88.67%. It is noteworthy that multiscale PE achieved a recognition rate of only 12.67% for motorboats, and multiscale DispEn achieved a recognition rate of only 58.00% for ocean noise. Overall, MTC-IPE-CP achieved an accuracy of 94.17%, which is 7.34% higher than multiscale DispEn and 19.17% higher than multiscale PE. These analyses results demonstrate the superior performance of the MTC-IPE-CP in accurately identifying and distinguishing between various types of ships.

## 5. Conclusions

In order to augment the discriminative power and noise robustness of entropy features in time series analysis, this paper introduces two novel time series analysis algorithms: TC-IPE-CP and MTC-IPE-CP. To validate the effectiveness of these methods, a comprehensive evaluation was conducted using both simulated signals and experimental signals. The simulation results indicate that TC-IPE-CP requires a small amount of data, exhibits strong noise resistance, and possesses high separability for signals. When applied to the analysis of heart rate signals, fault diagnosis, and underwater acoustic signal recognition, our experimental findings demonstrate that TC-IPE-CP can accurately differentiate between electrocardiographic signals of elderly and young subjects, achieve precise bearing fault diagnosis, and identify four types of underwater targets. Particularly in underwater acoustic signal recognition experiments, MTC-IPE-CP achieves a recognition rate of 96.67%, surpassing the well-known multi-scale dispersion entropy and multi-scale permutation entropy by 7.34% and 19.17%, respectively. This suggests that our method is highly suitable for the analysis of complex time series.

## Figures and Tables

**Table 1:** The correlation coefficients for generating AR processes.

	*α*_1_	*α*_2_	*α*_3_	*α*_4_	*α*_5_	*α*_6_	*α*_7_	*α*_8_
AR_1_	1/2	-	-	-	-	-	-	-
AR_2_	1/2	1/4	-	-	-	-	-	-
AR_3_	1/2	1/4	1/8	-	-	-	-	-
AR_4_	1/2	1/4	1/8	1/16	-	-	-	-
AR_5_	1/2	1/4	1/8	1/16	1/32	-	-	-
AR_6_	1/2	1/4	1/8	1/16	1/32	1/64	-	-
AR_7_	1/2	1/4	1/8	1/16	1/32	1/64	1/128	-
AR_8_	1/2	1/4	1/8	1/16	1/32	1/64	1/128	1/256

**Table 2:** Description of four types of ship-radiated noise.

Categories	Ship Name	Number of Segments
Passenger	Mar de Cangas	240
	Mar de Onza	130
	Pirata de Salvora	68
Ocean liner	MSC Opera	163
	Adventure of the Seas	95
	Costa Voyager	68
Motorboat	Small yacht	114
	Motorboat 2	123
	High speed motorboat	92
	Zodiac	99
Ocean noise	Natural ambient noise sample 1	85
	Natural ambient noise sample 2	99
	Natural ambient noise sample 3	98
	Natural ambient noise sample 4	93

**Table 3:** PNN classification results for four types of ships using MTC-IPE-CP features.

Categories	Ship Name				Classification Accuracy
	Passenger	Ocean Liner	Motorboat	Ocean Noise	
Passenger	145	5	0	0	96.67%
Ocean liner	0	150	0	0	100%
Motorboat	3	12	133	2	88.67%
Ocean noise	1	0	12	137	91.33%
In total	-	-	-	-	94.17%

**Table 4:** PNN classification results for four types of ships using multiscale PE features.

Categories	Ship Name				Classification Accuracy
	Passenger	Ocean Liner	Motorboat	Ocean Noise	
Passenger	131	0	0	19	87.33%
Ocean liner	0	150	0	0	100%
Motorboat	1	0	19	130	12.67%
Ocean noise	0	0	0	150	100%
In total	-	-	-	-	75.00%

**Table 5:** PNN classification results for four types of ships using multiscale DispEn features.

Categories	Ship Name				Classification Accuracy
	Passenger	Ocean Liner	Motorboat	Ocean Noise	
Passenger	141	9	0	0	94.00%
Ocean liner	0	150	0	0	100%
Motorboat	3	4	143	0	95.33%
Ocean noise	0	0	63	87	58.00%
In total	-	-	-	-	86.83%

## Data Availability

The data used to support the findings of this study are available from the corresponding author upon request.
